# Best practices and lessons learned from implementing a massive Ebola vaccination program: Summarizing UMURINZI team experience

**DOI:** 10.1002/hsr2.1618

**Published:** 2023-10-09

**Authors:** Roseline Dzekem Dine, Aline Uwizera Umutoni, Marie Michele Umulisa, Nnamdi Ezeanochie, Jozef Noben, Ellen Pagan Indoe, Clémence Dusingize, Fulgence Kamali, Julien Niyingabira

**Affiliations:** ^1^ Rinda Ubuzima Kigali Rwanda; ^2^ Johnson & Johnson New Brunswick New Jersey USA; ^3^ Janssen Global Public Health R&D Beerse Belgium; ^4^ Rwanda Biomedical Center and Rwanda Health Communication Center Kigali Rwanda

**Keywords:** best practices, community awareness, community engagement, Ebola, lessons learned, vaccine, western Rwanda

## Abstract

**Background and Aims:**

The unified Rwandan initiative for national ZEBOVAC immunization (UMURINZI) program's community engagement component was enacted to mobilize and vaccinate high‐risk community members. This article describes best practices and lessons learned from the implementation of UMURINZI, a large‐scale Ebola vaccination program.

**Methods:**

The population deemed to be at risk for EVD consisted of people who frequently cross Rwanda and the Democratic Republic of Congo (DRC) borders including those coming from Kigali City, potential first responders who have not previously been vaccinated against EVD, as well as people who reside in high‐risk border‐proximate areas of the Rubavu and Rusizi districts in the Western Province of Rwanda. These districts were selected because of their proximity to high‐traffic borders linking Rwanda to DRC's cities near an active Ebola outbreak. Volunteers of this program were adults, adolescents, and children aged 2 years or above who resided in the selected communities. Recruitment at the sites was conducted in close collaboration with each health area's Community Health Workers (CHWs). Volunteers were informed that the program involved being fully vaccinated (two doses of Ebola vaccines) within 2 months apart in the allocated vaccination sites.

**Results:**

Lessons learned were categorized into four pillars: infrastructure, leadership, myths, and partnership with respect. The best practices that were used during the implementation of the UMURINZI program were the results of a collaboration among CHWs, the involvement of national and local leaders, the use of a comprehensive engagement plan, and training. The study also had limitations.

**Conclusion:**

We described best practices and lessons learned during the implementation of the UMURINZI program in Rwanda. These practices and lessons learned represent promising options that could contribute to better community members' participation in mass vaccination programs. Hence, we demonstrated that rigorously designed community awareness and sensitization programs are effective for the implementation of similar programs in resource‐limited settings.

## 1 BACKGROUND

Comprehensive vaccination programs are the cornerstone of public health efforts and have led to the promotion of population health.^1^ Although well intended, poorly implemented vaccination programs have led to the waste of resources and/or limited abilities to use similar or the same methods in different settings or programs. Communicating and monitoring the implementation of public health programs is important to guide public policy decisions, however, not every program yields desired results because of poor implementation strategies that met the program setting.^2^ Strategies such as using and strengthening the local healthcare system, advocacy, community engagement, and monitoring and evaluation have been reported to improve participation in such programs.^1^, ^3^

The current literature supports six factors that improve program effectiveness and implementation. These factors include social networks, community awareness, partnership and collaboration, selection and training, provision of incentives, and political pressure/political will (including local leaders' recognition of the need for the program).^2^, ^4^, ^5^, ^6^ In Liberia, where an Ebola vaccine implementation clinical trial was initiated, limited infrastructure and an influx of volunteer participants led to site overcrowding.^3^ To overcome the problems they faced, capacity enhancement through a partnership also created room for the sharing of experience and dismissal of fears as well as myths about sound program implementation.^3^ During the implementation of the study entitled EBOVAC‐Salone: Lessons learned from implementing an Ebola vaccine trial in an Ebola‐affected country in Sierra Leone—Kambia, they faced difficulties engaging with the community especially due to limited country exposure to clinical trials and weak infrastructure which upon identification of those challenges, helped to design community engagement approaches and messaging for vaccine trial implementation.^7^

Discussion of the best practices and lessons learned from the implementation of the Unprecedented Movement to drive a unified Rwandan initiative for national ZEBOVAC immunization (UMURINZI) community engagement program opens the dialog to initiate similar large‐scale efforts in the future is vital. The program aimed to engage more than 200,000 community members to become fully vaccinated against the Ebola virus in the Rubavu and Rusizi districts of Rwanda.^8^, ^9^

## 2 AIM

Documentation of the best practices and lessons learned from the UMURINZI vaccination program is vital to inform policymakers as well as similar programs in Rwanda and globally. The purpose of this paper is to describe the identified best practices and lessons learned during the implementation of the 2‐year UMURINZI community engagement program within the Rubavu and Rusizi Districts of Rwanda.

## 3 METHODS

### 3.1 Program setting

The population deemed to be at risk for EVD consisted of people who frequently cross Rwanda and the Democratic Republic of Congo (DRC) borders including those coming from Kigali city, potential first responders who have not previously been vaccinated against EVD, as well as people who reside in high‐risk border‐proximate areas of the Rubavu and Rusizi districts in the Western Province of Rwanda. These districts were selected because of their proximity to high‐traffic borders linking Rwanda to DRC's cities near an active Ebola outbreak; this proximity indicated an increased likelihood of exposure to Ebola and therefore the greatest potential for vaccination against Ebola to contribute meaningfully to Ebola prevention in Rwanda.

### 3.2 Target population

The inclusion and exclusion criteria for this study are described below in Table 1.

**Table 1:** Description of study inclusion and exclusion criteria.

Inclusion criteria		Exclusion criteria	
Behavioral inclusion criteria	Crossing the border regularly	Medical exclusion criteria	Some people were not eligible to receive the vaccine based on medical conditions
	Working at or near border posts		Pregnancy
	Working at healthcare facilities in the target areas and/or vaccination sites		Less than 2 years of age
	Health facility staff (e.g., doctors, nurses, pharmacists, and cleaners)		Known allergies to vaccine ingredients for instance eggs
	UMURINZI program staff (Projet San Francisco/Center for Family Health Research [PSF/CFHR], Rinda Ubuzima [RU], and Janssen)		This was discussed individually between each potential volunteer and a nurse during prevaccination screening
	Other likely first responders in the event of active Ebola transmission (for instance, Red Cross volunteers and community health workers)		
Geographical inclusion criteria	UMURINZI began by targeting two districts of the Western Province of Rwanda (Rubavu and Rusizi), chosen because they share land borders with DRC. Geographical criteria were subject to potential expansion		

*Note*: The table shows the inclusion and exclusion criteria used during the UMURINZI community engagement program.

### 3.3 Volunteer recruitment description

Before the implementation of the UMIRINZI program, a community engagement guide that contained program sensitization, community mobilization, volunteers' recruitment, and follow‐up, as well as retention strategies, was developed by the Rwanda Biomedical Center, Rinda Ubuzima, and the Projet San Francisco team members. This tool was later approved by the Rwanda Biomedical Center because of its official role in managing all health data concerning citizens in Rwanda. With this tool, two approaches were used to recruit volunteers (2 years of age and above) with considerations for site specifications among both districts (targeted invitations and open enrollment). Targeted invitations were issued to border crossers at major border posts, target communities, and their potential contacts for instance family members and neighbors. Individuals who routinely crossed the border were encouraged to participate in the program. As UMURINZI progressed, vaccines were made available to all eligible individuals on a first‐come‐first‐served basis. Recruitment was conducted in close collaboration with each health facility area's community health workers (CHWs) as well as local authorities.

### 3.4 Implementation of recruitment

Ebola is a rare but dangerous virus associated with severe hemorrhagic fever that can lead to serious illness, including death. Due to the proximity of Rwanda to the DRC which has had several Ebola epidemics, the UMURINZI program was initiated. UMURINZI's program defines community engagement as “a systematic and ongoing process of working collaboratively with and through relevant community groups and partners.” The program was initiated in December 2019 to vaccinate community members with the Ad26.ZEBOV (first dose) and MVA‐BN®‐Filo (second dose) vaccines. UMURINZI's community engagement approach aimed to bring a supportive environment and positive behavior change amongst the communities at risk of an Ebola infection outbreak. It maximized opportunities for dialog, consultation, and formation of authentic partnerships and was mutually mindful and respectful of the social, cultural, and political realities of the individual communities alongside the border with DRC.

Specifically, the community engagement strategy aims to: mobilize the target population to receive vaccination; manage the recruitment process to ensure that the flow of potential vaccine recipients does not exceed daily per‐site capacity; and foster a conducive environment for voluntary participation in the vaccination campaign by addressing rumors, building community trust, and providing accurate information about risk and prevention strategies.

UMURINZI program collaborated with all stakeholder parties such as local government, community leaders, and public and private sectors to maximize the awareness, commitment, and participation of eligible citizens in the target area. The community engagement team used several best practices during the UMURINZI program while maintaining acceptable principles to foster trust in the community about the approach and the vaccine.

All vaccine recipients were informed that the full vaccine regimen requires two doses of the vaccine approximately 2 months apart. The first day of the campaign was launched with an introductory workshop and with 50 people invited for vaccination. Those offered to be vaccinated on that first vaccination day comprised well‐known, influential individuals from various groups in both districts. A list of invitees was first generated from various community leaders who had already expressed interest in vaccination, including leaders of associations; local government authorities; local health authorities; and others. All vaccine recipients received a health screening and had the opportunity to ask additional questions (and to opt out of vaccination, if desired) on launch day.

Mobilizers identified members of association members who met the program's behavioral inclusion criteria (routine border crossing and/or working at border sites) and thus were included in the program. Association‐level meetings were held to mobilize these groups for vaccination and to schedule appointments/issue invitations. Leaders of the association collaborated in the scheduling of members of the association to ensure a smooth and fair process. From these associations, and through active community engagement and outreach activities, community members became aware of the program and then became interested. People expressed interest in vaccination after being mobilized by voluntarily communicating when they were available to visit the vaccination sites. It was only after then that appointments were given. A list was generated for each day's appointments and those who arrived for vaccination were checked to ensure that they had an appointment for that day. However, following a thorough screening procedure, those considered “walk‐ins” were served as well.

The program started with few vaccination sites but as it scaled up, additional vaccination sites were opened in Rubavu and Rusizi districts.

### 3.5 Data collection

The method used for quantitative data collection during the UMURINZI program has been explained elsewhere by Nyombayire et al.^9^ However, different strategies including the use of volunteer reports, community feedback, interviews, surveys, or other methods of data collection such as door‐to‐door surveys obtained by CHWs were used for qualitative data collection. The combination of both types of data formed the base of this study.

## 4 RESULTS/FINDINGS

### 4.1 Lessons learned

Discussing lessons learned from the implementation of a successful program like the UMURINZI program's community engagement component is vital to inform policymakers as well as similar programs in Rwanda and the global community for the enhancement of global health practices aimed at bettering the population's health. These lessons learned are categorized into four main groups: infrastructure, leadership, myths, and partnership with respect.

#### 4.1.1 Infrastructure

UMURINZI program utilized brick‐and‐mortar buildings or tents to accommodate operations, staff, and volunteers. Sometimes, locations had a high daily attendance beyond expectations causing people to wait for long hours and could return to their homes without being vaccinated that day. To resolve this high influx of volunteers, more vaccination sites were established in targeted geographic locations, and the staff was deployed in those areas. These sites were established in collaboration with local government authorities to target the broader community who are eligible by the geographical inclusion criteria. These established vaccination sites might advance future vaccination programs within these districts. Limited space and distance to health facilities are commonly known as factors that make community members not uptake some health promotion programs, especially with the case of the Ebola vaccine whereas no case had been recorded in Rwanda at the time of the UMURINZI program. Hence, considerations for vaccination site capacity and methods to address the potential overflow of volunteers were key learnings that often are not being captured in most programs probably because of tight funding in the programs.

#### 4.1.2 Leadership

The engagement and visibility of leadership were key to the implementation of the UMURINZI community engagement program—the then Minister of Health launched the vaccination campaign in Rubavu district but also at Nkombo Island Health Center and the then Vice Minister of State in charge of Primary Healthcare, opened the vaccination campaign in Rusizi district. Visits by Wellcome Trust and Johnson & Johnson leaders were also scheduled while presentations to districts and province officials were also performed. In addition, health sector leaders were involved from the central level to the lower level (Ministry of Health, Rwanda Biomedical Center, District Hospitals, Health Centers, and CHWs). These key leaders' involvements created and maintained a supportive environment for citizens who were volunteering to receive the vaccine as they are trusted and believed to bring only what is good for the citizens. With the outbreak of COVID‐19, the attention of these leaders was divided, and they were forced to implement certain measures such as border closures and curfews among other restrictions. Potential UMURINZI volunteers were either caught up by curfews or by the limited numbers accepted at program sites. However, these leaders still played a key role in making room for the program to continue for instance allowing the program sites to halve the number of volunteers on sites in a day and issuing travel clearance for vehicles transporting volunteers to and from the vaccination sites. Depending on the context but unlike in Rwanda, if leaders such as health sector leaders are not involved in a program, community members are most likely not going to participate. Thus, having strong buy‐in from leadership was a key component to the success of this campaign, especially during a time of an unrelated communicable disease outbreak

#### 4.1.3 Myths

Myths arose within the community since the Ebola vaccination was a novel preventive option. For example, it was common to hear people saying that following the Ebola vaccination, volunteers will become infected with the Ebola virus. Efforts through mobilization from some program managers such as Rinda Ubuzima and their partners (for instance the Rwanda Biomedical Center and health providers) enabled volunteers to understand and reject those myths and rumors. In addition, the social mobilization used multiple communication approaches such as CHWs and community advisory groups that actively helped to mitigate some of the raised myths. Again, the fact that Rwandans normally have a high rate of vaccine acceptance, demand, and uptake as is the case with routine vaccines^10^ as well as the trust they have for their leaders including the Ministry of Health, might have influenced the dismissal of these myths. Thus, such comprehensive community engagement activities including existing trust, led to a positive turnout for the program. The need to acknowledge, evaluate, and mitigate myths about a novel vaccine was a key learning from the UMURINZI program.

#### 4.1.4 Partnership with respect

Stakeholder involvement during the implementation of the UMURINZI program was essential. The partnership created between Projet San Francisco and Rinda Ubuzima supported the awareness and sensitization of the program aimed at fully vaccinating more than 200,000 individuals. Both organizations during the program implementation were mutually mindful and respectful of the social, cultural, and political realities of the individual communities (for instance, before the beginning of the program), we engaged all leaders of associations (faith‐based leaders, CHWs, and teachers among others) and requested for best strategies to fully involve community members. One of the suggested and used strategies was recruiting program staff within the program communities. This aspect is sometimes forgotten in programs that make community members feel not valued and thus not adhere to program goals. Effective collaboration and communication during all stages of the program were vital to the program's success. They strive to respect volunteers at every step of the process, thus, cultivated a supportive environment for all volunteer participants.

### 4.2 Best practices

These were techniques that were experienced and proven reliable to lead the UMURINZI program to the desired results and were a collaboration with CHWs, involvement of national and local leaders, use of a comprehensive engagement plan, and training.

#### 4.2.1 Collaboration with CHWs

CHWs, after they received training on the UMURINZI program (with a focus on inclusion/exclusion criteria and daily vaccination site capacity) issued invitations to eligible community members. CHWs leveraged a variety of outreach strategies including village‐level mobilization meetings, meetings of associations/clubs in target areas, and home visits. CHWs mobilized volunteers by enumerating (counting/listing of people) community members, teaching about Ebola and the vaccination in community, and follow‐ups with volunteers to encourage retention. To ensure participant retention, CHWs reminded participants of the second dose (as they worked together with the vaccination manager, they were communicated with vaccination details of volunteers), and actively listened to and monitored participants during the implementation of the program. CHWs also responded to rumors (for instance by providing facts that they were taught about the disease and the vaccine to those concerned) to help boost the turnout at vaccination sites. This collaboration helped in the success of the program since CHWs in Rwanda are a trusted channel by community members within their respective communities.

#### 4.2.2 Involvement of national and local leaders

UMURINZI program in general collaborated from its start to the endpoint with different key persons such as government entities such as the Ministry of health both in Rwanda and the DRC and the Rwanda Biomedical Center that provided initial vaccination sites or existing health posts and CHWs. These government entities also laid a solid foundation for the implementation of the program by getting involved which served as a guarantee and a sign to community members that the program was trustable. Also, with the aid of local leaders (from the district level to the village level), meetings were organized to facilitate the flow of communication between the program and the community, hence increasing the population's understanding of the UMURINZI program benefits and uptake.

#### 4.2.3 Use of a comprehensive engagement plan

A couple of social networks were used during the UMURINZI program. All participants who were eligible for vaccination and volunteered to participate received the first dose and those with a mobile phone number were automatically enrolled into tracking systems such as the MOTECH mobile messaging platform unless they chose to opt‐out. This platform sent pre‐scheduled voice and SMS messages to remind participants of their upcoming second dose. The messages were timed to ensure adequate advanced notice of the second dose and provided contact information for the program's staff should they need to reach out to the vaccination program team. UMURINZI vaccination cards were given to each participant and used to fill out the date of their second dose appointments. The community needed to be sensitized extensively to maximize awareness and adherence to the vaccination program. As such, there were always group mobilizations where UMURINZI staff maintained communication with association leaders to ensure group‐level follow‐up of participants invited to the program. In addition to mass‐media communications, group‐based mobilization meetings were held with sub‐groups of the target population (e.g., faith‐based organizations, and parents' groups, among others). Sensitization messages emphasized that vaccination is voluntary, that all potential vaccine recipients were to be screened or tested for pregnancy (when applicable), and that they should expect to spend 45–90 min at the vaccination site. The community engagement coordinators prepared all materials needed for recruitment (tokens and banners, among others). Radio and television advertisements, megaphone announcements, billboards, and giant screens were broadcasted within target areas with sensitization messages.

#### 4.2.4 Training

The training was a very important aspect of the implementation of the UMURINZI program. For instance, the training (Rinda Ubuzima and Projet San Francisco staff) of trainers to be involved in community engagement and mobilization was a vital tool that facilitate the sound implementation of the program. The trained trainers further provided training to trainees (CHWs and healthcare providers). The training equipped them with all the information, education, communication materials, and processes required for successful community engagement. This method used for sensitization during the UMURINZI program helped to fully inform key persons and empowered them to help their community members deny certain myths such as the vaccine being a tool used by the government and foreigners “White people” to kill or send Rwandans to hell. Overall, training was essential to the empowerment of UMURINZI staff and volunteers to promote trust and confidence in the vaccination campaign.

#### 4.2.5 Resource mobilization

During the implementation of the UMURINZI community engagement program, several resources were mobilized including a formal documented community engagement plan. Flyers were produced for education, street banners pinned on strategic sites such as market places and bus stations, provision of remunerations (300 RWF) to program promoters (for instance, CHWs) depending on the number of volunteers they referred to the program site, radio, megaphones, television spots, billboards, brochures, and flip charts (given to health centers with information about the UMURINZI program). These helped create awareness of the program and ensured adherence to vaccination invitations. Furthermore, during this program, there was the outbreak of the COVID‐19 pandemic. As a result, transportation (boats, vehicles, motorbikes, and bus transportation), face masks, and soaps were provided to all volunteers to help them participate in the program while limiting the transmission of COVID‐19.

### 4.3 Study limitations

#### 4.3.1 Limited generalizability

The study's findings are based on the specific context of the UMURINZI program in Rwanda, targeting specific high‐risk populations in the border‐proximate areas, prone to active Ebola outbreaks. The effectiveness of the identified best practices may not be directly applicable to other regions or different infectious diseases.

#### 4.3.2 Sampling bias

The study mainly focuses on the participation of community members who reside in specific border‐proximate areas and may not fully represent the views or experiences of all community members, especially those living further away from the outbreak regions.

#### 4.3.3 Long‐term impact evaluation

While the study highlights a high adherence rate to the second dose invitation, it doesn't delve into the long‐term effectiveness of the vaccination in preventing Ebola outbreaks or mitigating their impact in the targeted communities.

## 5 CONCLUSION

We described the best practices and lessons learned during the community engagement of the UMURINZI program. A rigorously designed community awareness and sensitization program that gets community members and leadership involved as well as respects their values and cultures could be effective during the implementation of similar programs in any resource‐limited setting. While many lessons (infrastructure, leadership, myths, and partnership with respect) were learned from the community engagement of the UMURINZI program, some best practices (collaboration with CHWs, involvement of national and local leaders, use of a comprehensive engagement plan, and training) were utilized throughout, leading to 216,321 people adhering to the second dose invitations at the end of the program in September 2021 as described elsewhere (Community members' perspectives and adherence to invitations during the Ebola virus vaccination campaign in the Western Province of Rwanda). Although the lessons learned and best practices were specific to the context of the Ebola prevention strategy in Rwanda, some of the principles behind it may be transferable to similar mass community engagement programs.

## AUTHOR CONTRIBUTIONS

**Roseline Dzekem Dine**: Conceptualization; methodology; writing—original draft; writing—review and editing. **Aline Uwizera Umutoni**: Methodology; supervision; writing—original draft; writing—review and editing. **Marie Michele Umulisa**: Conceptualization; supervision; writing—original draft. **Nnamdi Ezeanochie**: Conceptualization; supervision. **Jozef Noben**: Conceptualization; supervision. **Ellen Pagan Indoe**: Supervision; writing—original draft; writing—review and editing. **Clémence Dusingize**: Supervision; writing—original draft. **Fulgence Kamali**: Conceptualization; supervision. **Julien Niyingabira**: Supervision; writing—review and editing.

## CONFLICT OF INTEREST STATEMENT

Nnamdi Ezeanochie, Jozef Noben, and Ellen Pagan Indoe are employees of Jassen as well as Johnson and Johnson. Other authors declare no conflict of interest.

## TRANSPARENCY STATEMENT

The lead author Roseline Dzekem Dine affirms that this manuscript is an honest, accurate, and transparent account of the study being reported; that no important aspects of the study have been omitted; and that any discrepancies from the study as planned (and, if relevant, registered) have been explained.

## ETHICS STATEMENT

While the UMURINZI community engagement program adhered to universal ethical principles, it received an ethical waiver from the Rwanda National Ethics Committee. All mobilized volunteers provided informed consent to participate.

## Data Availability

All consulted documents that formed the base of data for this study can be provided upon request from the corresponding author.

## References

[1] Hardt K , Bonanni P , King S , et al. Vaccine strategies: optimising outcomes. Vaccine. 2016;34(52):6691‐6699. 10.1016/j.vaccine.2016.10.078 27887796

[2] Durlak JA . The Importance of Quality Implementation for Research, Practice, and Policy. ASPE; 2013.

[3] Kennedy SB , Neaton JD , Lane HC , et al. Implementation of an Ebola virus disease vaccine clinical trial during the Ebola epidemic in Liberia: design, procedures, and challenges. Clin Trials. 2016;13(1):49‐56. 10.1177/1740774515621037 26768572

[4] Valente TW , Palinkas LA , Czaja S , Chu KH , Brown CH . Social network analysis for program implementation. PLoS One. 2015;10(6):e0131712. 10.1371/journal.pone.0131712 26110842 PMC4482437

[5] Silumbwe A , Zulu JM , Halwindi H , et al. A systematic review of factors that shape implementation of mass drug administration for lymphatic filariasis in sub‐Saharan Africa. BMC Public Health. 2017;17(1):484. 10.1186/s12889-017-4414-5 28532397 PMC5441010

[6] Downe S , Finlayson KW , Lawrie TA , et al. Qualitative evidence synthesis (QES) for guidelines: paper 1—using qualitative evidence synthesis to inform guideline scope and develop qualitative findings statements. Health Res Policy Syst. 2019;17(1):76. 10.1186/s12961-019-0467-5 31391057 PMC6686511

[7] Mooney T , Smout E , Leigh B , et al. EBOVAC‐Salone: lessons learned from implementing an Ebola vaccine trial in an Ebola‐affected country. Clin Trials. 2018;15(5):436‐443. 10.1177/1740774518780678 29895178 PMC6136072

[8] Karita E , Nyombayire J , Ingabire R , et al. Safety, reactogenicity, and immunogenicity of a 2‐dose Ebola vaccine regimen of Ad26.ZEBOV followed by MVA‐BN‐Filo in healthy adult pregnant women: study protocol for a phase 3 open‐label randomized controlled trial. Trials. 2022;23(1):513. 10.1186/s13063-022-06360-3 35725488 PMC9207821

[9] Nyombayire J , Ingabire R , Magod B , et al. Monitoring of adverse events in recipients of the 2‐dose Ebola vaccine regimen of Ad26.ZEBOV followed by MVA‐BN‐Filo in the UMURINZI Ebola vaccination campaign. J Infect Dis. 2023;227(2):268‐277. 10.1093/infdis/jiac283 35776140 PMC9833427

[10] National Institute of Statistics of Rwanda (NISR) [Rwanda] . Ministry of Health (MOH) [Rwanda], and ICF. 2020. Rwanda Demographic and Health Survey 2019‐20 Key Indicators Report. NISR and ICF.

